# Supplementary material for: Intra-Abdominal Hypertension and Compartment Syndrome after Pediatric Liver Transplantation: Incidence, Risk Factors and Outcome
Source: Children (Basel). 2022 Dec 18;9(12):1993. doi: 10.3390/children9121993 (PMC9777324; doi:10.3390/children9121993)
Supplement: Supplementary file 1 [file children-09-01993-s001.zip › children-1996448-supplementary.pdf]

# Supplement

Standard immunosuppressive regime:

- All patients one dose prednisolone during transplantation (300mg/square meter body surface)
- All patients Basiliximab on day 0 and 4 after pLT
- All patients long-term treatment with tacrolimus only (modigraf® from day 1)
    - except:
        - Patients with hepatoblastoma or below 6 kg bodyweight received Cyclosporine and Prednisolone as long term treament
        - Patient with combined lung-liver transplant: Tacrolimus, Prednisolone and Mycophenolate Mofetil.

Calculation of diuresis score:

**diuresis score** = hourly diuresis *[ml/kg/h]* **/** 1+ (furosemide*[mg/kg/d]* x 0.4 + etacrynic acid*[mg/kg/d]* x 0.1 + hydrochlorothiazide*[mg/kg/d]* x 0.04 + spironolactone*[mg/kg/d]* x 0.01 + mannitol*[g/kg/d]* x 0.13 + theophylline*[mg/kg/d]* x 0.01 + acetacolamide *[mg/kg/d]* x 0.005).

## Supplement Table S1

| Supplement Table 1: Graft and Transplant characteristics | | | | |
|---|---|---|---|---|
| | **No IAH** | **IAH** | **ACS** | **Total** |
| **Patients (n=)** | **4** | **6** | **17** | **27** |
| Type of LT: Living donor (LD) versus deceased donor | 75% LD | 17% LD | 18% LD | 26% |
| Size of transplant: whole organ versus sized-reduced whole organ versus split | 1 : 0 : 3(75%) | 4 : 0 : 2(33%) | 4 : 2 : 11(65%) | 9 : 2 : 16(59%) |
| Weight of explanted liver (g) * | 549.15 [±199.79] | 1064.90 [±565.30] | 743.61 [±765.70] | 787.84 [±670.28] |
| Weight of transplanted liver (g) * | 457.35 [±405.66] | 758.95 [±504.08] | 576 [±468.03] | 593.41 [±460.74] |
| Graft to-Recipient Weight Ratio * | 0.03 [±0.005] | 0.02 [±0.007] | 0.03 [±0.012] | 0.03 [±0,01] |
| Large for size transplantation | 1 (25%) | 0 | 5 (29%) | 6 (22%) |
| Duration of cold ischemia (min)* | 292.75 [±112.11] | 527.67 [±194.36] | 554.06 [±185.12] | 509.48 [±195.97] |
| Type of abdominal wall closure: primary versus sequential/staged | 1:3(75%) | 4:2(33%) | 2:15(88%) | 7:20(74%) |
| Aortic anastomosis of Arteria hepatica: yes versus no | 0:4(100%) | 2:4(66%) | 5:12(71%) | 7:20(0.74) |
| ***Mean [± standard deviation]** | | | | |

## Supplement Table S2

| | | | NoIAH* | | | | IAH * | | | | ACS* | | | | |
|---|---|---|---|---|---|---|---|---|---|---|---|---|---|---|---|
| **Parameter** | | **Unit** | Median [Min-Max] | Mean [Standard deviation] | Lower CI 95% | Upper CI 95% | Median [Min-Max] | Mean [Standard deviation] | Lower CI 95% | Upper CI 95% | Median [Min-Max] | Mean [Standard deviation] | Lower CI 95% | Upper CI 95% | p-value Kruskal Wallis |
| Heart frequency | | per minute | 100 [85-111] | 99 [±11] | 82 | 116 | 99 [90-128] | 103[±15] | 88 | 117 | 110[83-129] | 110[±15] | 103 | 119 | 0.25 |
| Mean arterial pressure (MAP) | | mmHg | 66[62-98] | 73[±17] | 46 | 100 | 92[63-96] | 81[±15] | 68 | 99 | 73[60-89] | 76[±11] | 70 | 80 | 0.34 |
| Near infrared spectroscopy (NIRS) | | % | 59 [56-64] | 59 [±6] | 6.6 | 112.1 | 75[71-89] | 79[±9] | 64.6 | 90.3 | 72[52-89] | 72[±11] | 65.8 | 78.3 | 0.14 |
| Dynamic Tissue Perfusion Measurement | Liver | cm/sec | 0.42[0.12-0.83] | 0.445[±0.363] | -0.13 | 1.02 | 0.30[0.09-0.45] | 0.310[±0.131] | 0.14 | 0.48 | 0.21[0.13-2.05] | 0.434[±0.509] | 0.13 | 0.76 | 0.96 |
| | Kidney | cm/sec | 0.17[0.9-0.42] | 0.211[±0.145] | -0.02 | 0.44 | 0.15[0.07-0.32] | 0.197[±0.126] | -0.01 | 0.35 | 0.27[0.1-2.05] | 0.394[±0.492] | 0.11 | 0.72 | 0.19 |
| | Spleen | cm/sec | 0.32[0.19-0.57] | 0.353[±0.181] | 0.07 | 0.64 | 0.42[0.26-0.55] | 0.376[±0.156] | 0.18 | 0.65 | 0.46[0.04-0.99] | 0.424[±0.304] | 0.23 | 0.61 | 0.96 |
| | Intestines | cm/sec | 0.07[0.04-0.36] | 0.135[±0.151] | -0.11 | 0.38 | 0.05[0.03-0.27] | 0.115[±0.138] | -0.08 | 0.29 | 0.10[0.01-0.29] | 0.115[±0.076] | 0.07 | 0.17 | 0.73 |
| Respiration frequency | | per minute | 28[26-31] | 28[±2] | 25 | 32 | 20[16-34] | 25[±8] | 16 | 32 | 24[14-46] | 27[±8] | 23 | 31 | 0.39 |
| D-GFR Score | | | 3.2[2.8-3.5] | 3.1[±0.3] | 2.7 | 3.7 | 2.4[1.0-3.0] | 2.1[±0.8] | 1.5 | 2.9 | 2.8[0.6-5.2] | 2.9[±1.2] | 2.3 | 3.5 | 0.10 |
| Albumin blood level | | g/dl | 24[21-25] | 23[±2] | 18 | 28.1 | 24[23-25] | 24[±1] | 22.2 | 26 | 24[18-32] | 25[±4] | 23 | 27.3 | 0.84 |
| Albumin substitution | | g | 3[2-10] | 4.5[±3.7] | -1.4 | 10.4 | 0[0-12] | 2.8[±5.2] | -2.7 | 7.4 | 0[0-18] | 1.8[±4.4] | -0.4 | 4.2 | 0.07 |
